# Detection, Characterization and Sequencing of BTV Serotypes Circulating in Cuba in 2022

**DOI:** 10.3390/v16010164

**Published:** 2024-01-22

**Authors:** Ana María Acevedo, Lydie Postic, Maray Curiel, Mathilde Gondard, Emmanuel Bréard, Stéphan Zientara, Fabien Vorimore, Mai-Lan Tran, Mathilde Turpaud, Giovanni Savini, Alessio Lorusso, Maurilia Marcacci, Damien Vitour, Pascal Dujardin, Carmen Laura Perera, Cristian Díaz, Yalainne Obret, Corinne Sailleau

**Affiliations:** 1 National Center for Animal and Plant Health (CENSA), Carretera de Tapaste y Autopista Nacional, Apartado Postal 10, San José de las Lajas, San José de las Lajas 32700, Cuba; acevedobeiras@gmail.com (A.M.A.); mara.oro18@gmail.com (M.C.); pereragonzalezc@gmail.com (C.L.P.); cristiandiaz9603@gmail.com (C.D.); obretferrery@gmail.com (Y.O.); 2 ANSES/INRAE/ENVA-UPEC, UMR 1161 Virology, Laboratoire de santé animale, 14 rue Pierre et Marie Curie, 94700 Maisons Alfort, France; lydie.postic@anses.fr (L.P.); mathilde.gondard@anses.fr (M.G.); emmanuel.breard@anses.fr (E.B.); stephan.zientara@vet-alfort.fr (S.Z.); mathilde.turpaud@anses.fr (M.T.); damien.vitour@vet-alfort.fr (D.V.); pascal.dujardin@vet-alfort.fr (P.D.); 3 Genomics Platform IdentyPath, Laboratory for Food Safety, ANSES, 94700 Maisons-Alfort, France; fabien.vorimore@anses.fr (F.V.); mai-lan.tran@anses.fr (M.-L.T.); 4 Istituto Zooprofilattico Sperimentale dell’Abruzzo e del Molise, 64100 Teramo, Italy; g.savini@izs.it (G.S.); a.lorusso@izs.it (A.L.); m.marcacci@izs.it (M.M.)

**Keywords:** BTV, serotypes, PCR, isolation, Sanger, Nanopore sequencing

## Abstract

In Cuba, despite a high sero-prevalence of bluetongue virus (BTV), circulating serotypes remain unknown. The aim of this study was to identify circulating BTV serotypes in farms throughout the western region of Cuba. Blood samples were collected from 200 young cattle and sheep between May and July 2022 for virological analyses (PCR, viral isolation and virus neutralization) and genome sequencing. The results confirmed viral circulation, with viro-prevalence of 25% for BTV. The virus was isolated from 18 blood samples and twelve BTV serotypes were identified by sequencing RT-PCR products targeting the segment 2 of the BTV genome (BTV-1, 2, 3, 6, 10, 12, 13, 17, 18, 19, 22 and 24). Finally, the full genome sequences of 17 Cuban BTV isolates were recovered using a Sequence Independent Single Primer Amplification (SISPA) approach combined to MinION Oxford Nanopore sequencing technology. All together, these results highlight the co-circulation of a wide diversity of BTV serotypes in a quite restricted area and emphasize the need for entomological and livestock surveillance, particularly in light of recent changes in the global distribution and nature of BTV infections.

## 1. Introduction

Bluetongue (BT) is an infectious, non-contagious disease caused by BTV which belongs to the *Orbivirus* genus of the *Sedoreoviridae* family [1]. This virus is transmitted by biting midges of the genus *Culicoides* (order Diptera, family *Ceratopogonidae*), mainly affecting domestic and wild ruminants [2].

BT is included on the WOAH list of notifiable diseases due to the rapid transmission and high dissemination of BTV, posing a risk of infection to various ruminant species [3]. The major negative impact of BTV infections lies in the economic losses due to international animal movement restrictions, as well as the disease prevention and control expenses affecting both individual farmers and the broader agricultural industry [4,5].

BTV has a genome of 10 double-stranded RNA segments (S) encoding 7 structural viral proteins (VP1 to VP7) and 6 non-structural proteins (NS1-NS4, NS3a and NS5) [6,7]. BTV has two capsid layers [8]: the outer capsid consists of VP2 and VP5, while VP7 and VP3 form the inner capsid which contains the viral genome and the replication complex (VP1, VP4 and VP6). VP2, the major constituent of the outer capsid, is exposed on the surface of the virus particle and determines the serotype-specific antigen.

Currently, 36 serotypes have been widely recognized [9,10,11,12,13]. The specific antigen (VP2) of each serotype induce the production of serotype-specific neutralizing antibodies.

Multiple serotypes have been identified in the Americas and Caribbean regions. Five BTV serotypes have been identified in North America, specifically BTV-10, 11, 13 and 17, while BTV-2 was restricted to the southeastern USA until 2010, when this serotype was reported in California [14]. Since 1998, 10 additional serotypes (BTV-1, 3, 5, 6, 9, 12, 14, 19, 22, 24), previously identified as exotic, have been isolated in the southeastern USA without being associated to any clinical cases [15,16,17]. In Central America and in the Caribbean islands, different BTV serotypes have been reported (serotypes 1, 2, 3, 4, 5, 6, 8, 9, 10, 11, 12, 13, 14, 17, 18, 22 and 24), although clinical signs have rarely been described in ruminants in these tropical and subtropical zones [2,18,19]. In South America, BTV has been reported in Argentina (serotype 4), Brazil (serotypes 4, 6, 14, 17, 19 and 20), Colombia (serotypes 12, 14 and 17), Chile, Ecuador (serotypes 9, 13 and 18), French Guiana (serotypes 1, 2, 6, 10, 12, 13, 17 and 24), Guyana (serotypes 14 and 17), Peru, Suriname (serotypes 6, 14 and 17) and Venezuela [18,20,21,22].

In Cuba, although the presence of BTV has been reported to the World Organization for Animal Health (WOAH) since 2007 and a large number of serologically positive animals have been reported [23], there is no evidence of clinical manifestations following BTV infections and the serotype(s) circulating are still unknown.

The objective of the present study was to identify the circulating serotypes of BTV from blood samples of cattle and sheep from different farms in the western region of Cuba. In addition, we present here the sequencing of 17 genomes of BTV strains from Cuba, using a Sequence Independent Single Primer Amplification (SISPA) approach—a version allowing Orbivirus sequences enrichment—and the Oxford nanopore MinION sequencing technology.

## 2. Materials and Methods

### 2.1. Study Area

Cuba is located in an area where BT is considered as endemic. Cuba is an archipelago with an area of 109,884 km^2^. The north is bordered by the state of Florida (USA). The average temperature (between 24 and 26 °C) and relative humidity (>60%) are high. The rainy period (May to October) records about 80% of the total annual rainfall. Cuba has a total of fifteen provinces and 168 municipalities, including the municipality of Isla de la Juventud. In this study, we selected two provinces of the western region of Cuba (Havana and Mayabeque) and four of its municipalities (Guanabacoa, San José de las Lajas, Catalina de Güines and Jaruco) to collect bovine samples.

### 2.2. Sampling Frame

**Figure 1:**
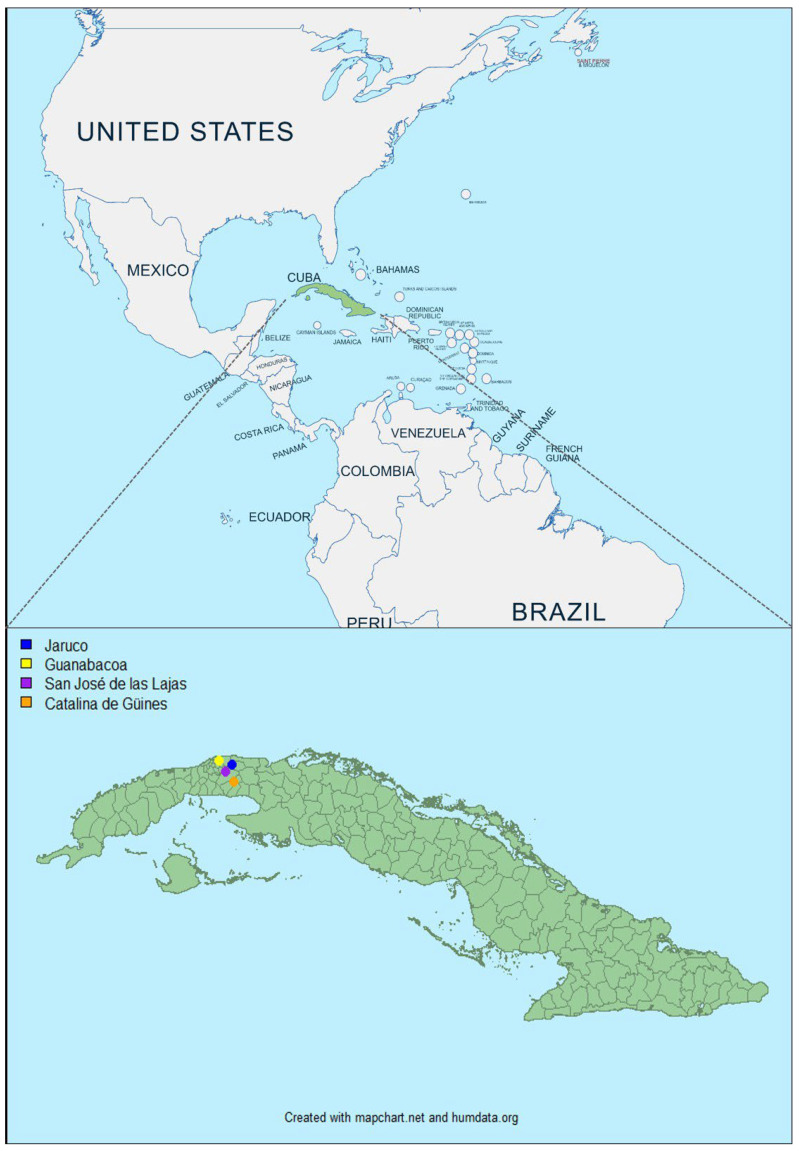
Geographical location of animals sampled in Cuba between May and July 2022. Colored dots indicate the collection sites of the blood samples. The dot color determines the municipalities: in Habana Province—Guanabacoa (Vista Hermosa, represented by a yellow dot) and in Mayabeque Province—San José de las Lajas (CENSA and Guayabal, represented by a pink dot), Catalina de Güines (ICA, represented by orange dot), Jaruco (Tenería 1 Perú, Típica 14 Perú and Típica 4 Perú, represented by blue dot).

Two-hundred blood samples were collected between May and July 2022 in seven distinct farms from Havana and Mayabeque, provinces from the western region of Cuba (Figure 1). They were obtained from young cattle (n = 183) and sheep (n = 17) in tubes containing ethylenediaminetetraacetic acid (EDTA) for virological assays. Animals were healthy and without clinical signs. These blood samples were stored at 4 °C for 8 to 10 months. They were sent at room temperature to the laboratory for animal health (Maisons Alfort, France). It took two weeks for the samples to arrive in France.

### 2.3. Molecular Analysis

#### 2.3.1. Nucleic Acid Sample Preparation

Total RNA was extracted from 100 µL of blood or cell culture supernatant using the Kingfisher 96 robot and the ID Gene™ Mag Universal Extraction Kit (Innovative Diagnostics, Grabels, France) according to manufacturer’s instructions. Finally, the RNAs were eluted with 80 µL of ultrapure water and used for BTV reverse-transcription polymerase chain reactions (RT-PCR).

#### 2.3.2. BTV Group-Specific Real-Time RT-PCR

Five µL of RNA denatured at 95 °C during 3 min were added to a commercial real-time RT-PCR (rtRT-PCR) kit mix (ADI-352, Bio-X Diagnostics S.A., Ploufragan, France) according to manufacturer’s instructions. This kit allowed all BTV serotypes to be detected, by amplification of a portion of the BTV S10 encoding NS3.

### 2.4. Virus Isolation

Viral isolations were first carried out on cultured Culicoides cells (KC cells) [24], then in the event of failure, blood was inoculated into embryonated eggs. Insect cells or eggs were inoculated with a 10^−1^ dilution of washed and lysed blood. However, when the hematite pellet (after washing the EDTA bloods) was tiny, the EDTA blood sample was simply inoculated diluted to the tenth.

#### 2.4.1. On KC Cells

A confluent monolayer of *Culicoides sonorensis* larvae cells (KC cells) was inoculated with diluted or lysed blood samples [24]. Inoculated flasks were incubated at 28 °C for 7 days. One hundred µL of culture cell supernatant was analyzed by BTV rtRT-PCR. If the cycle threshold (Ct) value was >30, a second passage was carried out. If the CT was <30, the cells/supernatants were inoculated to BSR cells following the protocol described in Section 2.4.3.

#### 2.4.2. On Embryonated Chicken Eggs

Groups of three embryonated chicken eggs (ECE) were each intravenously inoculated with 0.1–0.2 mL of diluted or lysed BTV RT-PCR-positive blood samples [25]. The eggs were incubated until 7 days at 35 °C and examined daily. The embryos that died between days 2 and 7 were removed and homogenized. The tissue homogenates were then clarified by centrifugation at 2000× *g* for 10 min at 4 °C and 100 µL of the supernatant was tested by RT-PCR.

#### 2.4.3. On BSR Cells

Positive RT-PCR supernatants from KC or homogenized embryonated eggs were inoculated into BSR cells. Inoculated flasks were incubated at 37 °C and examined under a microscope every day for 7 days to check for cytopathic effects (CPE).

### 2.5. BTV Serotyping Using Subgroup-Specific RT-PCR and Sanger Sequencing

BTV serotypes of the eighteen Cuban strains successfully isolated were then determined using conventional RT-PCR and Sanger sequencing of the genomic S2. Eight primer pairs, designed to specifically target the different BTV nucleotypes forming by the clustering of the S2 sequences of the 24 classic BTV serotypes, were used in conventional RT-PCR using the one-step RT-PCR Kit (Qiagen, France) as already described [21,26]. Five microliters of each RT-PCR products was analyzed by electrophoresis on a 1.5% agarose gel and directly sequenced in both directions, using the primer pairs used for amplification (Eurofins Genomics, Ebersberg, Germany). Sanger sequencing results were assembled using Geneious Prime (version 2022.0.2) and compared to the homologous sequences available in GenBank using the online BLAST search tool.

### 2.6. Virus Neutralization Test

Virus neutralization test (VNT) was carried out using methods similar to those described in the WOAH Manual of Standards for Diagnosis tests and vaccines [27]. Constant amounts of each BTV type-specific antiserum (50 µL/well) were added in microtiter plates to a tenfold dilution series of each virus sample (50 µL/well) and the serum–virus mixtures incubated for 1 h at 37 °C. BSR cells (100 µL/well), diluted in Eagle’s MEM, supplemented with 10% of fetal calf serum, 100 µg/µL streptomycin and 100 IU/mL penicillin, were then added at a concentration of 10^5^ cells/mL. Microtiter plates were sealed and incubated at 37 °C for 7 days. The BTV type-specific antiserum that reduced the virus titre by at least two log_10_ compared with the virus control in the absence of any antiserum designated the virus serotype.

### 2.7. Minion Sequencing

The 17 Cuban strains of BTV isolated on BSR cells or embryonated chicken eggs were sequenced using a SISPA approach [28,29] adapted to orbiviruses sequencing and the Oxford Nanopore MinION technology.

#### 2.7.1. RNA Extraction and Validation

Total RNA was extracted from 140 µL of cell culture virus using a QIAamp Viral RNA Mini Kit and QIACube automat (Qiagen, Hilden, Germany) according to the manufacturer’s instructions (without addition of RNA carrier). Extracted RNA was eluted into 60 μL of nuclease-free water and 5 µL of RNA (denatured 95 °C; 3 min) was analyzed using a commercial pan-BTV kit as described below (ADI-352, Bio-X Diagnostics, Ploufragan, France).

#### 2.7.2. cDNA Synthesis and Amplification Using the SISPA Approach

First, overrepresented rRNA from the host were depleted using the NEBNext^®^ rRNA Depletion Kit with RNA Sample Purification Beads (New England Biolabs, Ipswich, MA, USA) according to manufacturer’s instructions. Then, ds cDNAs were produced and amplified using a SISPA approach; a combination of random-tagged primers (FR26RV-N; 50 mM), and specific-tagged primers (FR-BT_F; 10 mM and FR-BT_R; 10 mM) targeting the conserved extremities of the ten orbiviruses genomic segments (see Table 1). Briefly, ds RNA samples were denatured at 95 °C during 5 min and set at 4 °C for 3 min. Then, RNAs were reverse transcribed into cDNA with Transcriptase inverse SuperScript™ IV Kit (Life Technologies, Carlsbad, CA, USA) according to the manufacturer’s instructions. Second-strand synthesis (SSB) of the cDNA was performed by adding 1 µL (5 U) of polymerase, Klenow Fragment (3′→5′ exo-,) (New England Biolabs, USA), at 37 °C, 60 min and 10 min at 75 °C. A tenfold dilution of the ds cDNA was then amplified using the FR20_Rv primer targeting the SISPA tag (see Table 1) and the Q5^®^ Hot Start High-Fidelity DNA Polymerase (New England Biolabs, USA) as recommended. Produced amplicons were purified using HighPrep PCR Clean-up System (MagBio Genomics Inc., Gaithersburg, MD, USA) and eluted in 30 µL of DNase free water. Finally, total ds cDNA was quantified with the dsDNA High Sensitivity (HS) assay Kit and Orbivirus ds cDNA checked using BTV specific rtRT-PCR (without the RT step). The quality and the average size of the amplicon sample was assessed using a TapeStation 2200 system (Agilent Technology, Santa Clara, CA, USA) and the Genomic DNA ScreenTape kit (Agilent Technologies, USA).

#### 2.7.3. Sequencing Using Oxford Nanopore Technology

Sequencing libraries were prepared using the SQK-LSK109 Kit and EXP-NBD104 or EXP-NBD114 Native Barcode expansion (Oxford Nanopore Technologies, Oxford, UK) following the ligation sequencing amplicons protocol with native barcoding available on the manufacturer website (version “NBE_9065_v109_revAP_14Aug2019”). Fifty fmoles of final pooled library were loaded onto a FLO-MIN106 R9.4.1 flow cell (ONT). A 72 h run was conducted with standard settings and the MinKNOW software (version 22.12.7).

### 2.8. Sequences Data Analysis

#### 2.8.1. Sequencing Data Analysis

Raw reads were basecalled and demultiplexed using GUPPY (version 6.4.6) and the highly accurate model. Then, reads were analyzed using a custom mapping workflow on Geneious Prime (version 2022.0.2). First, SISPA labels were removed from the read sequences (20 bases removed in 5′ and 3′ of each read), size selection was applied in order to select reads with expected length (between 150 and 9000 bp). Trimmed and filtered reads were then mapped on reference sequences using minimap2 (kmer length of 10) selected according to BTV serotype and geographic origin (BTV-1: OP185814 to OP185823; BTV-2: OP185734–OP185743; BTV-3: MT815674–MT815683; BTV-10: MZ395202–MZ395211; BTV-12: OP185744–OP185753; BTV-13: KY049872–KY049880 and KX442584; BTV-17: OR611816–OR611825; BTV-18: KY049881–KY049889 and KX442585; BTV-19: MT815694–MT815703; BTV-22: OP185864–OP185873; BTV-24: OP185764–OP185773). Consensus sequences were produced with a minimum of 100 of sequencing depth. In a few cases, if the mapping quality allowed it, the minimum of sequencing depth was lowered to recover the conserved segment extremities. Genomes were annotated and deposited in GenBank (see Section 3.4 for accession number).

#### 2.8.2. Phylogenetic Analyses

The identification of the closest nucleotide homology available in GenBank nt database was performed for each genomic segment using the online BLAST search tool. Alignment and phylogenetic analysis were performed using MEGA X (version 10.2.0) [32]. Alignments were achieved using MUSCLE [33], and phylogenetic trees reconstructed using the Maximum Likelihood method and Tamura–Nei model, with a bootstrap of 1000 [34]. The trees were drawn to scale, with branch lengths established by measuring in the number of substitutions per site. All positions containing gaps and missing data were eliminated. Further information is provided in the figure legends.

## 3. Results

### 3.1. Pan-BTV Real-Time RT-PCR Analyses

A total of 50 out of 200 (25%) blood samples tested in this study were found positive for BTV using the pan-BTV RT-qPCR system, with Ct values ranging from 22 to 38. Among the positive blood samples, 45 were collected from bovine and five from sheep.

### 3.2. Viral Isolation

Forty-three blood samples were selected for viral isolation (BTV-positive blood samples with Ct values inferior to 33), and 18 BTV isolates were obtained. Thirteen were obtained with KC cells and five with embryonated chicken eggs (Table 2).

### 3.3. Serotype Determination

The serotypes of the 18 BTV isolates were determined by conventional RT-PCR, using subgroup-specific primers, and Sanger sequencing of the PCR products. The partial S2 sequences obtained were identified by alignment with sequences available in GenBank. A total of 12 BTV serotypes were identified (BTV-1, 2, 3, 6, 10, 12, 13, 17, 18, 19, 22 and 24) (Table 2). Almost all isolates corresponded to mono-infection, but one sample was found co-infected by BTV-6 and 22. Serotype identification was also confirmed by the VNT analysis, except for the co-infected strain for which VNT was not possible.

### 3.4. Full Genome Sequencing

The genome of the 17 mono-infected BTV isolates were fully recovered using the SISPA-MinION sequencing approach described in this study, including the genome of one BTV-2, 3, 13, 19, 22 and 24 strain, two BTV-1, 10, 12 and 17 strains and three BTV-18 strains (Table 3).

### 3.5. Phylogenetic Analysis of S2 (VP2) and S6 (VP5) of the Cuban BTV Strains

Sequences of S2 and S6, encoding the outer capsid proteins, were fully recovered and annotated. Closest homologies between the Cuban sequences and established sequences were determined using blastn algorithm and Genbank database (Table 4). S2 sequences recovered from Cuba displayed 95 to 98% of identity with published sequences from the western hemisphere, mostly sequences from USA and some others from French Guiana (Table 4). Similar homologies results were observed with the alignment of Cuban S6 sequences, except for serotype 3, which only displayed 93% of identity with a reference sequence from USA.

**Figure 2:**
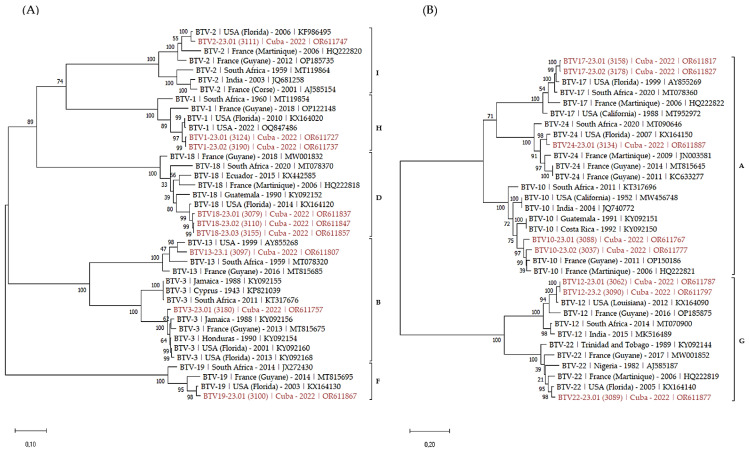
Phylogenetic analysis of S2 sequences of the Cuban BTV strains. Phylogenetic analysis of S2 sequences of BTV-1, 2, 3, 13, 18, 19 (**A**), 10, 12, 17, 22 and 24 (**B**) strains using the maximum likelihood method and Tamura–Nei model with 1000 bootstrap replicates in MEGA X. This analysis involved 39 (**A**) and 33 (**B**) nucleotide sequences, and there were a total of 2932 (**A**) and 2997 (**B**) positions in the final dataset. Bootstrap values appeared at the corresponding nodes. In the phylogenetic tree, accession number, serotype, country and year of sample collection are given. The Cuban sequences are marked in red. Brackets indicate nucleotypes clustering.

**Figure 3:**
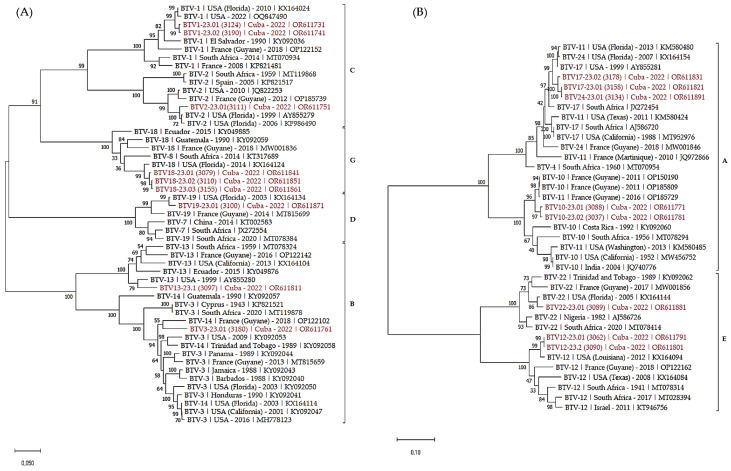
Phylogenetic analysis of S6 sequences of the Cuban BTV strains. Phylogenetic analysis of S6 sequences of BTV-1, 2, 3, 13, 18, 19 (**A**), 10, 12, 17, 22 and 24 (**B**) strains using the maximum likelihood method and Tamura–Nei model with 1000 bootstrap replicates in MEGA X. This analysis involved 51 (**A**) and 37 (**B**) nucleotide sequences, and there were a total of 1581 (**A**) and 1645 (**B**) positions in the final dataset. Bootstrap values appeared at the corresponding nodes. In the phylogenetic tree, accession number, serotype, country and year of sample collection are given. The Cuban sequences are marked in red. Brackets indicate nucleotypes clustering.

Finally, phylogenetic trees of S2 (Figure 2) and S6 (Figure 3) were built to analyze the phylogenetic relationship between the Cuban sequences and sequences in Genbank. We selected both closely related and more distant sequences from the eastern hemisphere to add some diversity.

As expected, S2 sequences clustered according to the serotype of the BTV strains. Within each serotype, Cuban sequences clustered with sequences identified in the western hemisphere, including USA, Latin America and the Caribbean. As well, serotypes clustering followed the nucleotypes grouping [35,36].

The analysis of S6 sequences confirmed most of the previous observations. The majority of the sequences clustered according to nucleotypes and the serotypes.

Sequence homology research results on the seven other genomic segments (1, 3, 4, 5, 7, 8, 9 and 10) of the 18 BTV strains are available in the Supplementary Materials (Table S1), together with distance matrices and phylogenetic trees resulted from the alignments of the Cuban sequences for each segment (Figures S1–S8 and Table S2).

## 4. Discussion

Blood samples analyzed in this study were collected on asymptomatic animals from a quite small area including four municipalities in the province of Havana and Mayabeque. Yet, prevalence of BTV among our sampling reached 25% (50 PCR BTV positive blood samples out of 200 tested). Together with a previous work conducted 10 years ago that demonstrated 99.7% of BTV seropositivity among 1100 healthy bovines, our results strongly support the endemicity of BTV on the island [23]. Scant epidemiological data are available on BTV life cycle in the area; however, multiple parameters could explain the high prevalence of the virus, including the presence of the Culicoides vector, such as Culicoides insignis [15,37], the absence of vector control measures or BTV surveillance, and the asymptomatic circulation of the virus.

Eighteen Cuban BTV strains were successfully isolated from 43 blood samples (those with required viral load). This good yield of virus isolation highlights that despite the delay between sample collection and the analyses, in addition with the substandard storage conditions and transport, blood samples infectivity was not altered. Indeed, blood samples were stored for 6 to 8 months at +4 °C, and then at room temperature for around 3 weeks (including time spent in Havana and during air transport to France). Lysing and washing the blood before isolation on KC cells or eggs and then, in case of failure, inoculating EDTA blood diluted to the 10th, helped to the success of the viral isolation tests. Finally, as a last attempt, five more isolates were obtained on embryonated eggs.

This study is the first characterization of the BTV serotypes circulating on the island of Cuba. All isolates were serotyped using classical RT-PCRs and Sanger sequencing of the segment 2 and confirmed by VNT. Surprisingly, a total of twelve BTV serotypes (1, 2, 3, 6, 10, 12, 13, 17, 18, 19, 22 and 24) were identified, showing a high level of co-circulation of BTV strains in a very restricted area close to Havana, at least between May and July 2022. It would be very interesting to investigate other regions of Cuba to find out whether additional serotypes are circulating and if contrasted situation could be observed on the island. Serotypes identified in this study were also reported in different regions of the Americas such as the USA, Latin America and the Caribbean, including the Lesser Antilles, an area close to Cuba, suggesting a large distribution within the western hemisphere [2,14,18,21].

In this study, we also presented for the first time the recovery of BTV full genomes from Cuba using the combined approach of SISPA sample preparation and MinION sequencing. The twelve BTV serotypes were fully confirmed. Most of the S2 and S6 recovered from strains belonging to the same serotype shared very high nt homology (from 98.4 to 99.8%), forming the same phylogenetic cluster. In contrast, S2 of the two BTV-10 strains displayed only 93.6% of nt identity and each of the Cuban strains were more closely related to BTV-10 strains from French Guiana (94.9 and 95.7%), suggesting that two different BTV-10 are circulating in this region.

While S2 and S6 analysis are fundamental for orbiviruses serotyping, the recovery of the eight other segments offer the opportunity to obtain some insights into the relationships and dynamic of BTV strains. Alignments of the Cuban S1, S3, S4, S5, S7, S8, S9 and S10 displayed very different clustering patterns, all completely independent from the BTV serotypes identified, highlighting multiple reassortment phenomena between the different segments and strains (Supplementary Data). Interestingly, some segments displayed remarkable nucleotide diversity (below 96% of nt identity) but quite conserved protein sequences, such as VP7, with twelve isolates sharing a similar sequence (100% of aa identity), additionally to VP1 and VP3 displaying 98.5 to 100% and 99.4 to 100% of AA homology, respectively. The predominance of these protein sequences could suggest that they represent the best adaptation to their environment and hosts (insect or mammal) [38].

Finally, the closest homology and phylogenetic analysis of the Cuban BTV segments supported a common origin with the North American strain. Indeed, most of the S2 and S6 from Cuba shared 92.6 to 98.4% of nt identity with strains from the USA, and similar observations were made when analyzing the closest homology of the other segments (Supplementary Data Figures S1–S8 and Table S1). Particularly, homology research of the Cuban S9 even suggested recent exchanges in the area with closest homology implying American strains described in 2022 (Supplementary Data Table S1). Although database could influence at some extent such results, considering that the USA and France overseas department are the main sources of western BTV genomes submitted in Genbank, the geographical proximity of these two countries might explain the close phylogenetic relationship between the Cuban and the American strains. Bluetongue, like other vector-borne diseases, can be introduced into new regions through legal and illegal movements of susceptible hosts or through conveyance of the vector in vehicles or by the wind, or through the exchange of infected semen or embryos [39]. The proximity of Cuba to the southern region of the USA could be a possible route of exchange of BTV serotypes by windborne transportation of infected Culicoides [40]. The closest distance between Cuba and the USA is Key West, the main island of the Keys, located 90 miles (144 km) south of Florida. Even though the vectors that transmit BTV have a limited flight time, they can be easily transported over long distances by the wind [41], even as far as 700 km under certain climatologic conditions when wind speed is sufficient [42] and orographic barriers are absent [43,44].

Collectively, the results of this study underline the great BTV diversity circulating in Cuba where the virus seemed well established. More epidemiological studies are required to better understand BTV life cycle in those (sub)-tropical area, such as vectors and hosts involved, in both domesticated and wild life, BTV distribution and ways of exchange between the Caribbean islands and the Americas. To conclude, in addition to the importance of serotyping in disease surveillance and control, more efforts should be made in BTV genotypes characterization and genome sequencing to decipher BTV evolution and dynamic.

## Figures and Tables

**Table 1:** Primers used for cDNA synthesis and amplification.

Primers	Sequences 5′-3′	Reference
FR26RV-N (50 µM)	GCCGGAGCTCTGCAGATATCNNNNNNN	[29]
FR-BT_F (10 µM)	GCCGGAGCTCTGCAGATATCGTTAAAN	[30,31]
FR-BT_R (10 µM)	GCCGGAGCTCTGCAGATATCGTAAGTN	[30,31]
FR20RV (40 µM)	GCCGGAGCTCTGCAGATATC	[29]

**Table 2:** BTV serotypes identified in Mayabeque and Havana provinces.

Province	Municipality	Identified BTV Serotype (Isolate Number)
Mayabeque	San José de las Lajas	2 (1), 10 (1), 12 (1), 13 (1), 18 (1), 19 (1), 22 (1)
	Catalina de Guïnes	10 (1), 12 (1), 18 (1)
	Jaruco	1 (1), 3 (1), 17 (2), 18 (1), 24 (1)
Havana	Guanabacoa	1 (1), 6/22 (1)

**Table 3:** Corresponding accession numbers of the Cuban strain genomic sequences deposited on NCBI.

Cuban Strain	Accession Number (S1–10)
BTV1-23.01 (3124)	OR611726–OR611735
BTV1-23.02 (3190)	OR611736–OR611745
BTV2-23.01 (3111)	OR611746–OR611755
BTV3-23.01 (3180)	OR611756–OR611765
BTV10-23.01 (3088)	OR611766–OR611775
BTV10-23.02 (3037)	OR611776–OR611785
BTV12-23.01 (3062)	OR611786–OR611795
BTV12-23.2 (3090)	OR611796–OR611805
BTV13-23.1 (3097)	OR611806–OR611815
BTV17-23.01 (3158)	OR611816–OR611825
BTV17-23.02 (3178)	OR611826–OR611835
BTV18-23.01 (3079)	OR611836–OR611845
BTV18-23.02 (3110)	OR611846–OR611855
BTV18-23.03 (3155)	OR611856–OR611865
BTV19-23.01 (3100)	OR611866–OR611875
BTV22-23.01 (3089)	OR611876–OR611885
BTV24-23.01 (3134)	OR611886–OR611895

**Table 4:** First closest homology between theS2 and S6 sequences of the Cuban strains and sequences available in GenBank with 100% of sequence coverage (AN: accession number; Id%: percentage of nucleotide identity).

Cuban Strain	Seg.	AN	Length (bp)	Closest Homology	Closest Homology AN	Id %	Origin	Date
BTV1-(3124)	2	OR611727	2940	BTV 1 USA/FL 10-044273 segment 2	KX164020	98.0	USA	2010
	6	OR611731	1635	BTV 1 USA/FL 10-044273 segment 6	KX164024	98.4	USA	2010
BTV1-(3190)	2	OR611737	2940	BTV 1 USA/FL 10-044273 segment 2	KX164020	98.0	USA	2010
	6	OR611741	1635	BTV 1 USA/FL 10-044273 segment 6	KX164024	98.4	USA	2010
BTV2-(3111)	2	OR611747	2943	BTV 2 USA VP2 gene	KF986495	98.0	USA	2006
	6	OR611751	1635	BTV 2 FL99 13406-2 VP5 protein gene	AY855279	97.7	USA	1999
BTV3-(3180)	2	OR611757	2935	BTV 3 USA/FL 138555-30 L2 VP2 gene,	KY092160	97.7	USA	2001
	6	OR611761	1637	BTV 3 USA2001/FL 138555-30 M6 VP5 gene	KY092047	92.6	USA	2001
BTV10-(3088)	2	OR611767	2926	BTV 10 (3937) VP2 gene	OP150186	94.9	GUY	2011
	6	OR611771	1638	BTV 10 (3937) segment 6	OP150190	97.0	GUY	2011
BTV10-(3037)	2	OR611777	2926	BTV 10 (3937) VP2 gene	OP150186	95.7	GUY	2011
	6	OR611781	1638	BTV 10 (4138) segment 6	OP185809	96.9	GUY	2011
BTV12-(3062)	2	OR611787	2904	BTV 12 USA/LA 12-046093 segment 2	KX164090	97.3	USA	2012
	6	OR611791	1645	BTV 12 USA/LA 12-046093 segment 6	KX164094	97.9	USA	2012
BTV12-(3090)	2	OR611797	2904	BTV 12 USA/LA 12-046093 segment 2	KX164090	97.3	USA	2012
	6	OR611801	1645	BTV 12 USA/LA 12-046093 segment 6	KX164094	97.8	USA	2012
BTV13-(3097)	2	OR611807	2935	BTV 13 FL99 22364-8 VP2 protein gene	AY855268	94.6	USA	1999
	6	OR611811	1637	BTV 13 FL99 22364-8 VP5 protein gene	AY855280	94.6	USA	1999
BTV17-(3158)	2	OR611817	2923	BTV 17 FL99 12475 VP2 protein gene	AY855269	96.0	USA	1999
	6	OR611821	1638	BTV 17 FL99 12475 VP5 protein gene	AY855281	96.4	USA	1999
BTV17-(3178)	2	OR611827	2923	BTV 17 FL99 12475 VP2 protein gene	AY855269	95.9	USA	1999
	6	OR611831	1638	BTV 17 FL99 12475 VP5 protein gene	AY855281	96.6	USA	1999
BTV18-(3079)	2	OR611837	2927	BTV 18 USA/FL 15-008010 segment 2	KX164120	97.7	USA	2014
	6	OR611841	1637	BTV 18 USA/FL 15-008010 segment 6	KX164124	97.7	USA	2014
BTV18-(3110)	2	OR611847	2927	BTV 18 USA/FL 15-008010 segment 2	KX164120	97.9	USA	2014
	6	OR611851	1637	BTV 18 USA/FL 15-008010 segment 6	KX164124	97.5	USA	2014
BTV18-(3155)	2	OR611857	2927	BTV 18 USA/FL 15-008010 segment 2	KX164120	98.0	USA	2014
	6	OR611861	1637	BTV 18 USA/FL 15-008010 segment 6	KX164124	97.4	USA	2014
BTV19-(3100)	2	OR611867	2938	BTV 19 USA/FL 280559-3 segment 2	KX164130	98.1	USA	2003
	6	OR611871	1637	BTV 19 USA/FL 280559-3 segment 6	KX164134	98.3	USA	2003
BTV22-(3089)	2	OR611877	2907	BTV 22 USA/FL 402286 segment 2	KX164140	97.0	USA	2005
	6	OR611881	1645	BTV 22 USA/FL 402286 segment 6	KX164144	97.6	USA	2005
BTV24-(3134)	2	OR611887	2923	BTV 24 USA/FL 520518 segment 2	KX164150	96.4	USA	2007
	6	OR611891	1638	BTV 17 FL99 12475 VP5 protein (S5) gene	AY855281	96.2	USA	1999

## Data Availability

All data presented in this study are summarized in the paper. The detailed data of this study are available on request from the corresponding author.

## Supplementary Materials

The following supporting information can be downloaded at: https://www.mdpi.com/article/10.3390/v16010164/s1, Figures S1–S8: Phylogenetic analysis of the Cuban strains of BTV, segments 1, 3, 4, 5, 7, 8, 9 and 10; Table S1: First closest homology between the genomic sequences of the Cuban strains and reference sequences available in GenBank with 100% of sequence coverage (AN: accession number of the recovered sequence; Id% and Cov% percentage of nucleotide identity and coverage between Cuban and reference sequences); Table S2: Distance matrices determined from the Cuban sequences alignment.
