# Supplementary material for: Detection, Characterization and Sequencing of BTV Serotypes Circulating in Cuba in 2022
Source: Viruses. 2024 Jan 22;16(1):164. doi: 10.3390/v16010164 (PMC10819738; doi:10.3390/v16010164)
Supplement: Supplementary file 1 [file viruses-16-00164-s001.zip › Cuba_paper_figures_S1_S8_sup_data.pdf]

Supplementary Figures. Phylogenetic analysis of the Cuban strains of BTV, segments 1, 3, 4, 5, 7, 8, 9 and 10.

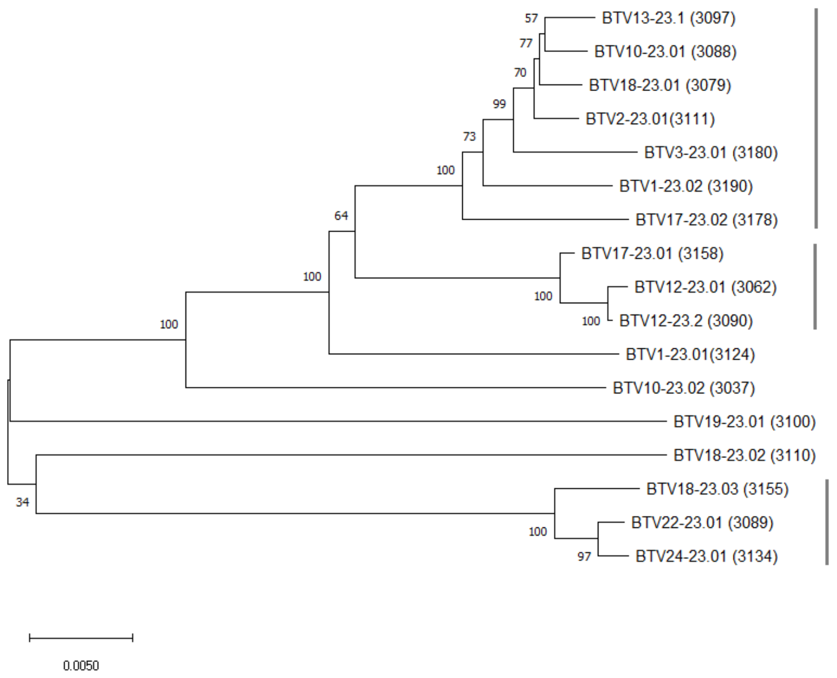

Figure S1: Phylogenetic analysis of S1 sequences of the Cuban BTV strains. Phylogenetic analysis of S1 sequences of BTV strains using the Maximum Likelihood method and Tamura-Nei model (1000 replicates). This analysis involved 17 nucleotide sequences; there were a total of 3944 positions in the final dataset. In the phylogenetic tree, GenBank sequences, species designations and strain names are given. Brackets include sequences sharing more than 98% of nucleotide identity.

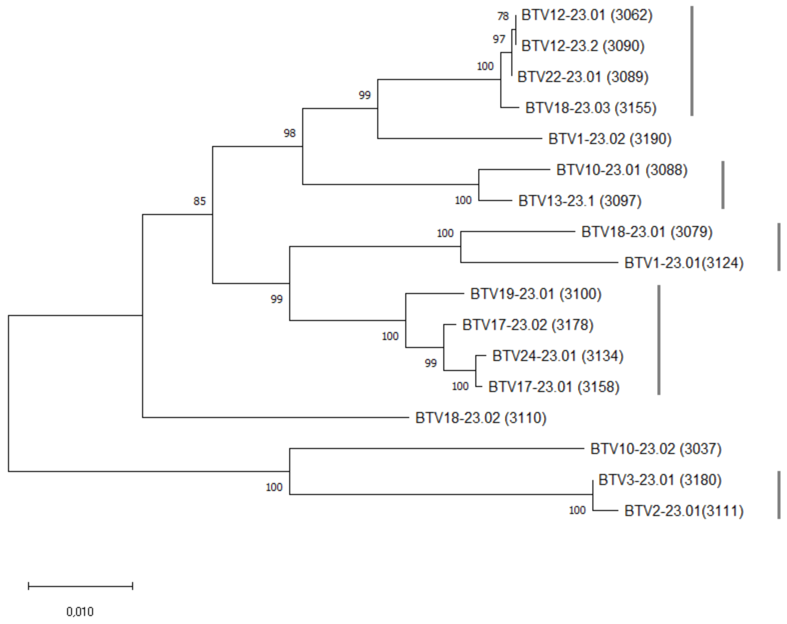

Figure S2: Phylogenetic analysis of S3 sequences of the Cuban BTV strains. Phylogenetic analysis of S1 sequences of BTV strains using the Maximum Likelihood method and Tamura-Nei model (1000 replicates). This analysis involved 17 nucleotide sequences, there were a total of 2772 positions in the final dataset. In the phylogenetic tree, GenBank sequences, species designations and strain names are given. Brackets include sequences sharing more than 98% of nucleotide identity.

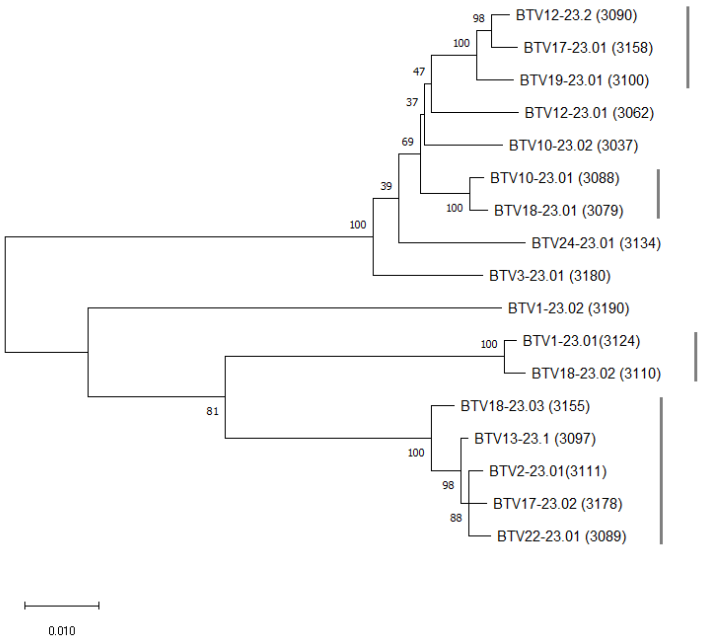

Figure S3: Phylogenetic analysis of S4 sequences of the Cuban BTV strains. Phylogenetic analysis of S4 sequences of BTV strains using the Maximum Likelihood method and Tamura-Nei model (1000 replicates). This analysis involved 17 nucleotide sequences, there were a total of 1981 positions in the final dataset. In the phylogenetic tree, GenBank sequences, species designations and strain names are given. Brackets include sequences sharing more than 98% of nucleotide identity.

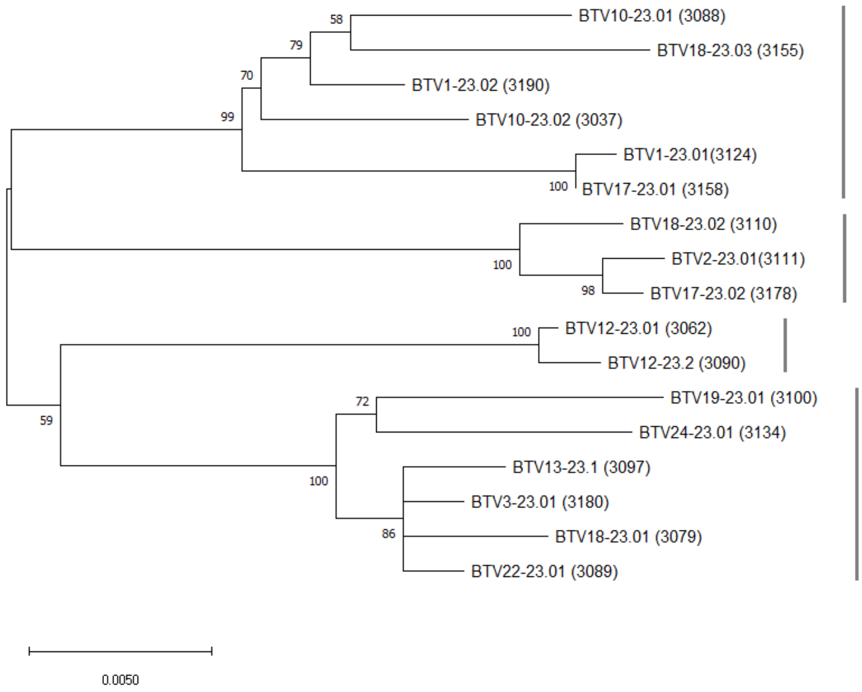

Figure S4: Phylogenetic analysis of S5 sequences of the Cuban BTV strains. Phylogenetic analysis of S5 sequences of BTV strains using the Maximum Likelihood method and Tamura-Nei model (1000 replicates). This analysis involved 17 nucleotide sequences, there were a total of 1771 positions in the final dataset. In the phylogenetic tree, GenBank sequences, species designations and strain names are given. Brackets include sequences sharing more than 98% of nucleotide identity.

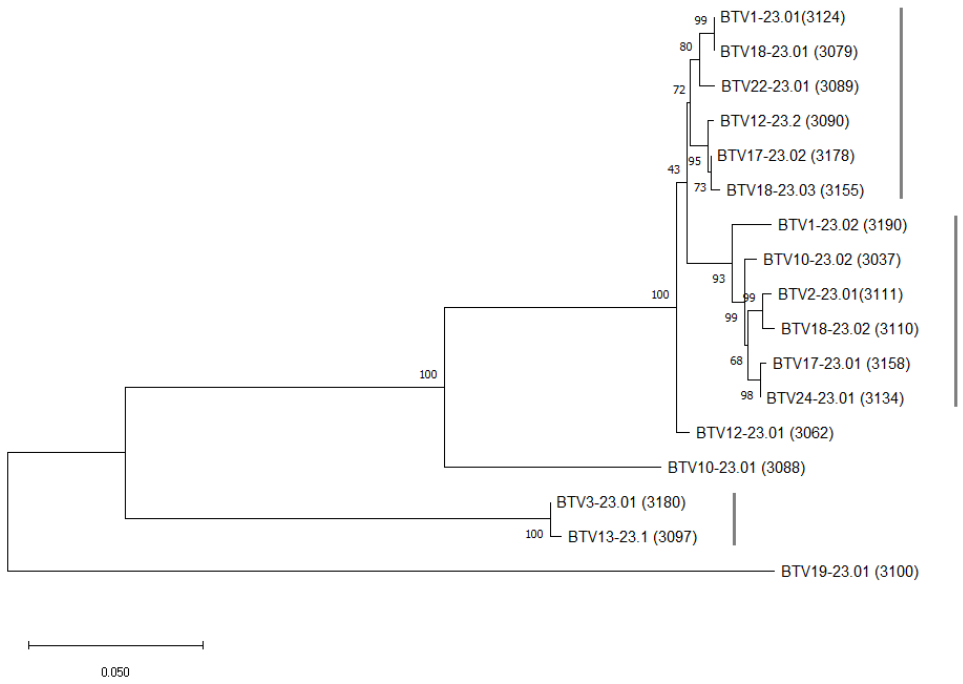

Figure S5: Phylogenetic analysis of S7 sequences of the Cuban BTV strains. Phylogenetic analysis of S8 sequences of BTV strains using the Maximum Likelihood method and Tamura-Nei model (1000 replicates). This analysis involved 17 nucleotide sequences, there were a total of 1159 positions in the final dataset. In the phylogenetic tree, GenBank sequences, species designations and strain names are given. Brackets include sequences sharing more than 98% of nucleotide identity.

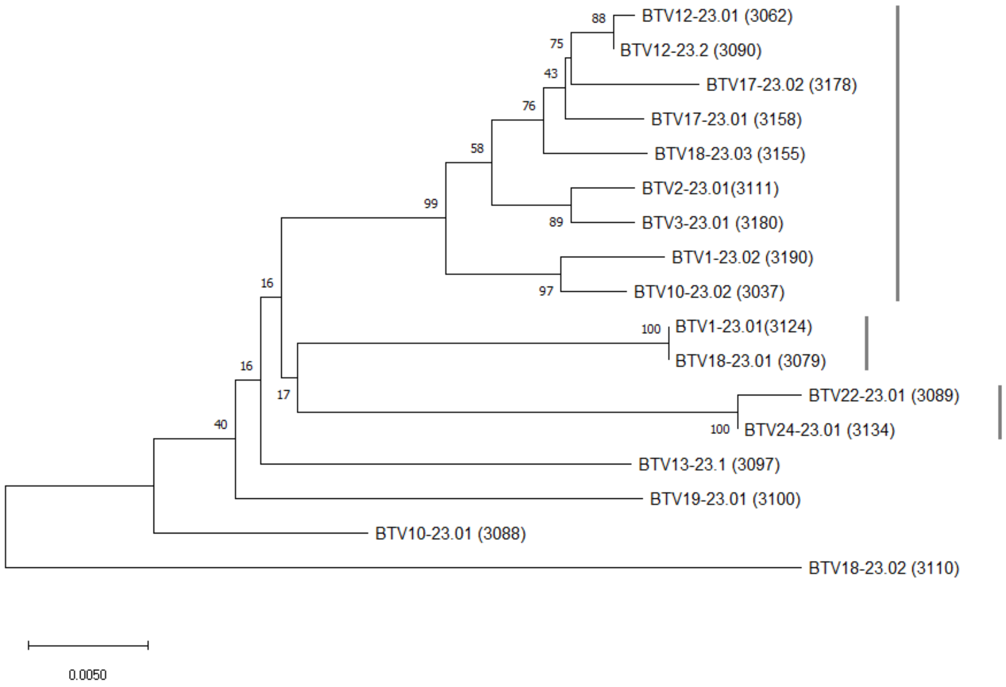

Figure S6: Phylogenetic analysis of S8 sequences of the Cuban BTV strains. Phylogenetic analysis of S8 sequences of BTV strains using the Maximum Likelihood method and Tamura-Nei model (1000 replicates). This analysis involved 17 nucleotide sequences, there were a total of 1125 positions in the final dataset. In the phylogenetic tree, GenBank sequences, species designations and strain names are given. Brackets include sequences sharing more than 98% of nucleotide identity.

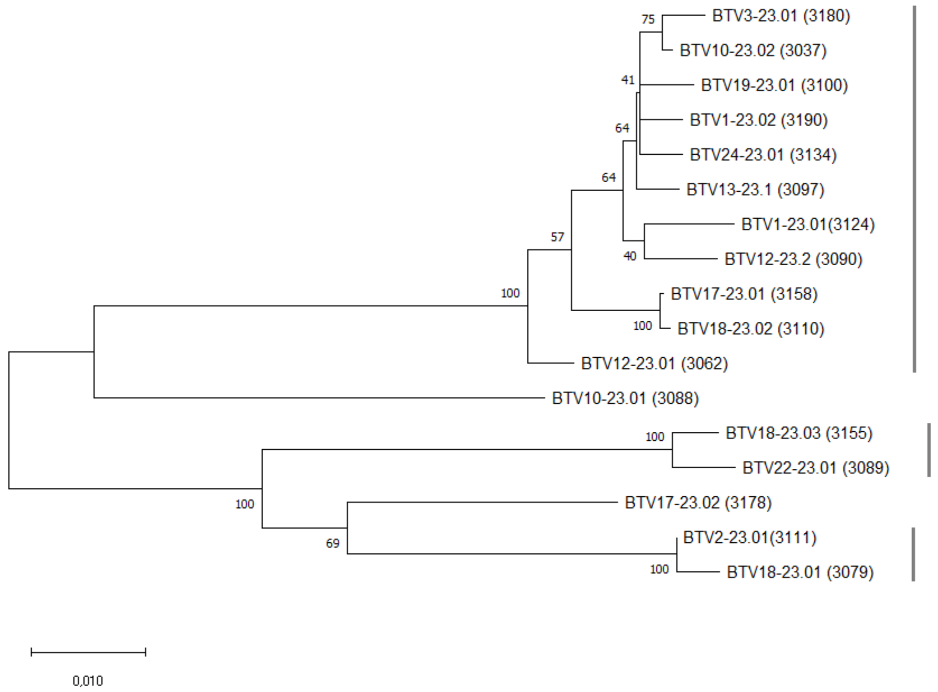

Figure S7: Phylogenetic analysis of S9 sequences of the Cuban BTV strains. Phylogenetic analysis of S8 sequences of BTV strains using the Maximum Likelihood method and Tamura-Nei model (1000 replicates). This analysis involved 17 nucleotide sequences, there were a total of 1050 positions in the final dataset. In the phylogenetic tree, GenBank sequences, species designations and strain names are given. Brackets include sequences sharing more than 98% of nucleotide identity.

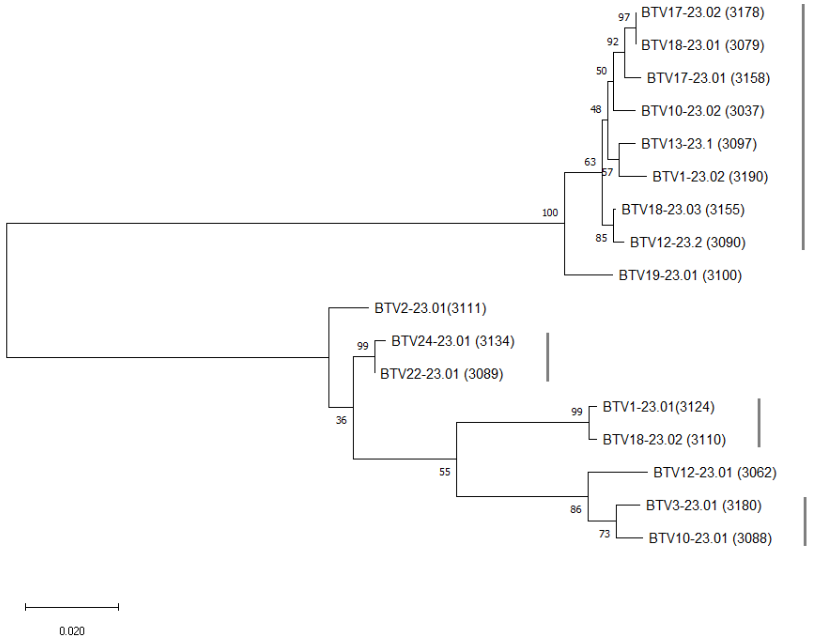

Figure S8: Phylogenetic analysis of S10 sequences of the Cuban BTV strains. Phylogenetic analysis of S8 sequences of BTV strains using the Maximum Likelihood method and Tamura-Nei model (1000 replicates). This analysis involved 17 nucleotide sequences, there were a total of 822 positions in the final dataset. In the phylogenetic tree, GenBank sequences, species designations and strain names are given. Brackets include sequences sharing more than 98% of nucleotide identity.
